# Pulsed Electromagnetic Fields Stimulate HIF-1α-Independent VEGF Release in 1321N1 Human Astrocytes Protecting Neuron-like SH-SY5Y Cells from Oxygen-Glucose Deprivation

**DOI:** 10.3390/ijms21218053

**Published:** 2020-10-28

**Authors:** Fabrizio Vincenzi, Silvia Pasquini, Stefania Setti, Simona Salati, Ruggero Cadossi, Pier Andrea Borea, Katia Varani

**Affiliations:** 1 Department of Morphology, Surgery and Experimental Medicine, Section of Pharmacology, University of Ferrara, 44121 Ferrara, Italy; psqslv@unife.it (S.P.); vrk@unife.it (K.V.); 2 Igea Biophysics Laboratory, 41012 Carpi, Italy; s.setti@igeamedical.com (S.S.); s.salati@igeamedical.com (S.S.); r.cadossi@igeamedical.com (R.C.); 3 University of Ferrara, 44121 Ferrara, Italy; bpa@unife.it

**Keywords:** pulsed electromagnetic fields, VEGF, astrocytes, astrocyte-conditioned medium

## Abstract

Pulsed electromagnetic fields (PEMFs) are emerging as an innovative, non-invasive therapeutic option in different pathological conditions of the central nervous system, including cerebral ischemia. This study aimed to investigate the mechanism of action of PEMFs in an in vitro model of human astrocytes, which play a key role in the events that occur following ischemia. 1321N1 cells were exposed to PEMFs or hypoxic conditions and the release of relevant neurotrophic and angiogenic factors, such as VEGF, EPO, and TGF-β1, was evaluated by means of ELISA or AlphaLISA assays. The involvement of the transcription factor HIF-1α was studied by using the specific inhibitor chetomin and its expression was measured by flow cytometry. PEMF exposure induced a time-dependent, HIF-1α-independent release of VEGF from 1321N1 cells. Astrocyte conditioned medium derived from PEMF-exposed astrocytes significantly reduced the oxygen-glucose deprivation-induced cell proliferation and viability decrease in the neuron-like cells SH-SY5Y. These findings contribute to our understanding of PEMFs action in neuropathological conditions and further corroborate their therapeutic potential in cerebral ischemia.

## 1. Introduction

Biophysical stimulation employing pulsed electromagnetic fields (PEMFs) is widely used in orthopedic practice to promote bone healing and joint chondroprotection [1]. However, emerging evidence has identified PEMFs as an attractive non-invasive strategy also for the treatment of different neuropathological conditions. In particular, the effects of PEMF stimulation have been studied in the context of Parkinson’s disease [2,3], Alzheimer’s disease [4], and neuropathic pain [5]. Moreover, a promising target for the potential therapeutic intervention with PEMFs seems to be cerebral ischemic diseases. In a rabbit model of transient focal ischemia, PEMF exposure attenuated cortical ischemia edema and reduced ischemic neuronal damage [6]. More recently, following distal middle cerebral artery occlusion in mice, PEMFs significantly reduced neuroinflammation and pro-apoptotic factors and determined a reduction of infarct size, implicating PEMFs as possible adjunctive therapy for stroke patients [7]. In mice subjected to photothrombotic occlusion, PEMFs increased pro-survival proteins, while decreased pro-apoptotic proteins and pro-inflammatory mediators activating the BDNF/TrkB/Akt signaling pathway [8]. In a previous study, we reported the protective effect of PEMFs on hypoxia and inflammation damage in neuron-like and microglial cells [9]. We then investigated the signaling pathway that underlies the anti-apoptotic and pro-survival effect of PEMFs in neuron-like cells subjected to hypoxia through the activation of p38 kinase cascade enrolling HSP70, CREB, BDNF, and regulating the Bcl-2 family proteins [10] and the anti-inflammatory effect of PEMFs in microglial cells [11]. 

Despite much information that have been collected in neurons and microglial cells, little is known about the effect of PEMFs on astrocytes. In the healthy central nervous system, astrocytes are involved in the regulation of neurodevelopment, neurotransmission, cerebral metabolism, and blood flow [12,13]. In conditions of neuronal injury, such as stroke, astrocytes protect neurons against oxidative stress and excitotoxicity [14]. In in vitro and in vivo models of brain ischemia, astrocytes are generally more resistant and better preserved than neurons in the boundary zone to the infarct [15,16]. In addition to protecting neurons from oxidative stress and excitotoxicity, the surviving astrocytes could help to re-establish the neuronal function in the ischemic penumbra [17]. In response to ischemia, astrocytes produce multiple neurotrophic factors to protect neurons and restoring brain homeostasis by promoting neurogenesis and angiogenesis [18]. During ischemic conditions, numerous studies report the protective effect of vascular endothelial growth factor (VEGF) which can directly exert pro-survival effects in neurons and may promote reparative mechanisms through its angiogenic effects. In an in vitro model of ischemia, VEGF secreted in astrocyte conditioned medium (ACM) enhances Akt-enabled cell survival signaling in neurons through activation of VEGF receptor-2 leading to less neuronal cell death [19]. VEGF dose-dependently enhanced the survival of rat cortical neurons by decreasing active caspase-3 levels and increasing expression of the anti-apoptotic protein Bcl-2 [20]. Neuroprotective effects of VEGF have also been demonstrated in various in vivo models of stroke. Local application of VEGF to the surface of the reperfused brain reduced the infarction volume in a rat middle cerebral artery occlusion model of transient ischemia [21]. In another study, intracerebroventricular administration of VEGF reduced infarct size, improved neurological performance, enhanced the delayed survival of newborn neurons and stimulated angiogenesis in the striatal ischemic penumbra [22]. In addition to its established function in erythropoiesis, erythropoietin (EPO) is considered a potential therapeutic strategy for various types of brain injuries. Systemic administration of single or multiple doses of recombinant EPO was shown to confer neuroprotection in several animal models of brain ischemia [23]. In vitro, EPO secreted into ACM increased neuron cell viability and inactivated pro-apoptotic proteins [24]. It has been proposed that transforming growth factor-beta 1 (TGF-β1) released by astrocytes could help preserve brain function during the subacute period after stroke limiting neuronal cell death [25,26].

In order to clarify the mechanism of PEMF-induced protective effect in different models of cerebral ischemia, the purpose of this study was to investigate the influence of PEMFs on the release of key neurotrophic factors from 1321N1 astrocytes. Furthermore, the effect of ACM derived from PEMF-exposure 1321N1 cells was evaluated on cell proliferation and viability decrease in neuron-like cells induced by oxygen-glucose deprivation.

## 2. Results

### 2.1. Effect of Pulsed Electromagnetic Fields on Cells Viability and Neurotrophic Factor Production in 1321N1 Astrocytes

**Figure 1:**
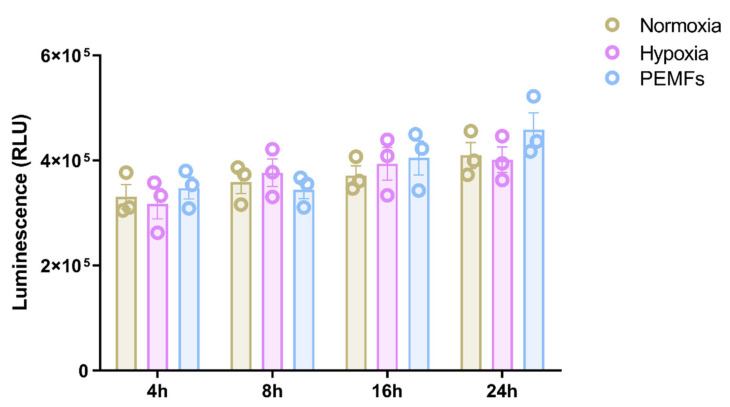
1321N1 cell viability in conditions of normoxia, hypoxia or PEMF exposure in normoxia. 1321N1 cells were incubated from 4 to 24 h in normoxia in the absence or presence of PEMFs and hypoxic conditions. Cell viability was assessed by measuring intracellular ATP content. Luminescence values expressed as relative light unit (RLU) and proportional to intracellular ATP content are reported as an index of SH-SY5Y cell viability. Data are expressed as the mean ± SEM of three independent experiments.

The effect of PEMFs was investigated on the production of VEGF, EPO and TGFβ1, well-known neurotrophic and neuroprotective factors released from glial cells in response to hypoxic, excitotoxic, or metabolic injury. The potential effect of PEMF on the release of neurotrophic factors was evaluated in 1321N1 cells in comparison to hypoxic conditions. Firstly, to exclude a possible effect of PEMFs on cell viability, 1321N1 astrocytes were exposed to PEMFs or hypoxic conditions for 4–24 h. At all time points investigated, neither PEMFs nor hypoxia affected 1321N1 cell viability (Figure 1).

**Figure 2:**
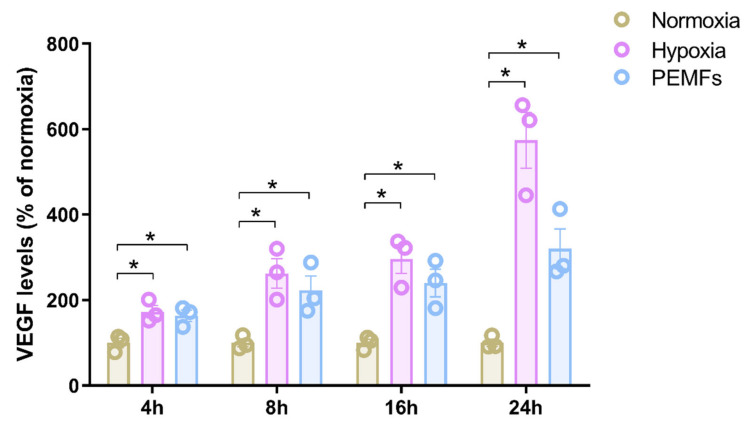
VEGF released by 1321N1 following normoxia, hypoxia or PEMF treatment in normoxia. 1321N1 cells were incubated from 4 to 24 h in normoxia in the absence or presence of PEMFs and hypoxic conditions. Supernatant was collected for the quantification of VEGF levels. Data are expressed as mean ± SEM of three independent experiments. *, *p* < 0.01 vs. normoxia at the same time period.

The effect of PEMFs on the release of VEGF, one of the most important angiogenic and neurotrophic factors, was then investigated. Since it is well-known that hypoxia stimulates the release of VEGF expression and secretion, the effect of PEMF exposure was compared with hypoxic conditions for different exposure times, from 4 to 24 h. As shown in Figure 2, hypoxia determined a significant increase of VEGF release at all the time points investigated. The maximum increase was obtained after 24 h of incubation in hypoxic conditions where VEGF production augmented from 288 ± 25 to 1624 ± 189 pg/mL (*p* < 0.01). Interestingly, PEMF exposure similarly increased VEGF release from 1321N1 astrocytes in a time-dependent manner (Figure 2). Even in this case, the maximum effect was obtained at 24 h of incubation were PEMF exposure induced a 3.2-fold increase of VEGF production, from 288 ± 25 to 922 ± 134 pg/mL (*p* < 0.01).

**Figure 3:**
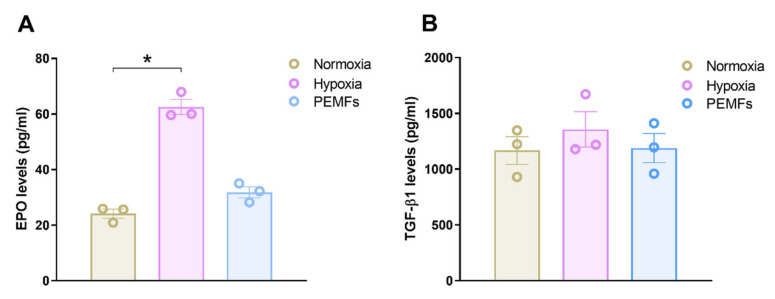
EPO and TGF-β1 production by 1321N1 following normoxia, hypoxia or PEMF treatment in normoxia. (**A**) EPO levels measured in 1321N1 medium after 24 h of incubation in normoxia, hypoxia or PEMF exposure during normoxia. (**B**) Histograms depicting TGF-β1 concentration in 1321N1 medium after 24 h of incubation in normoxia, hypoxia or PEMF exposure during normoxia. Data are expressed as mean ± SEM of three independent experiments. *, *p* < 0.01 vs. normoxia.

The effect of PEMFs was then evaluated on the production of EPO and TGFβ1. After 24 h of incubation, hypoxic conditions induced a significant EPO release in 1321N1 cells, with an increase of 2.6 fold with respect to the control condition (Figure 3A). PEMF exposure did not modulate EPO expression in 1321N1 cells, suggesting a different response from hypoxia. Regarding TGF-β1, neither PEMF exposure nor hypoxia modulated TGFβ1 release from 1321N1 cells (Figure 3B).

### 2.2. The PEMF-Induced Release of VEGF in 1321N1 Cells Is Not Mediated by HIF-1α

**Figure 4:**
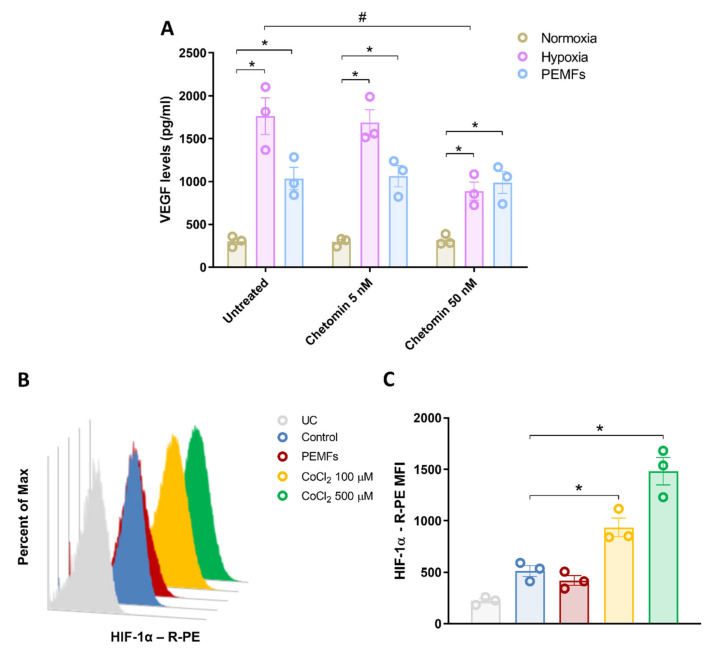
Evaluation of HIF-1α involvement in the effect of PEMFs. (**A**) 1321N1 cells treated or untreated with the HIF-1α inhibitor chetomin (5 and 50 nM) were incubated in normoxia, hypoxia, or in the presence of PEMFs during normoxia and VEGF were quantified after 24 h of incubation. Data are expressed as mean ± SEM of three independent experiments. *, *p* < 0.01 vs. normoxia. #, *p* < 0.01 vs. untreated cells subjected to hypoxia. (**B**) Representative histogram plot overlay of HIF-1α-R-PE fluorescence intensity in 1321N1 cells exposed for 24 h to PEMFs or CoCl_2_ (100 and 500 µM, used as a chemical inducer of HIF-1α). (**C**) Median fluorescence intensity (MFI) of R-PE conjugated HIF-1α antibody in 1321N1 cells exposed for 24 h to PEMFs or CoCl_2_ (100 and 500 µM). Data are expressed as the mean ± SEM of three independent experiments. *, *p* < 0.01 vs. control.

Since VEGF release in hypoxic conditions is generally mediated by the activation of HIF-1α, we tested the hypothesis that PEMF-induced VEGF release in 1321N1 cells was related to the activity of this transcription factor. To this aim, the effect of PEMF exposure for 24 h on VEGF release was evaluated in the absence and the presence of the HIF-1α inhibitor chetomin at two different concentrations (5 and 50 nM). The presence of 5 nM chetomin did not affect VEGF release (Figure 4A). As expected, cell treatment with chetomin at the 50 nM concentration significantly inhibited hypoxia-mediated VEGF release from 1321N1 cells. On the contrary, the PEMF-mediated increase of VEGF release was not affected by the presence of chetomin, even at the 50 nM concentration (Figure 4A). 

To further corroborate the HIF-1α-independent action of PEMFs on 1321N1 astrocytes, HIF-1α expression following 24 h PEMF exposure was investigated using flow cytometry. As a control positive, the chemical inducer of HIF-1α CoCl_2_ was used at the 100 and 500 µM concentrations. In 1321N1 cells, PEMFs exposure did not modulate HIF-1α expression confirming that the PEMF-mediated VEGF production was independent by the activation of this transcriptional regulator of cellular response to hypoxia (Figure 4B,C).

### 2.3. Astrocyte Conditioned Medium Derived from PEMF-Exposed 1321N1 Cells Protects SH-SY5Y Cells from Oxygen-Glucose Deprivation

**Figure 5:**
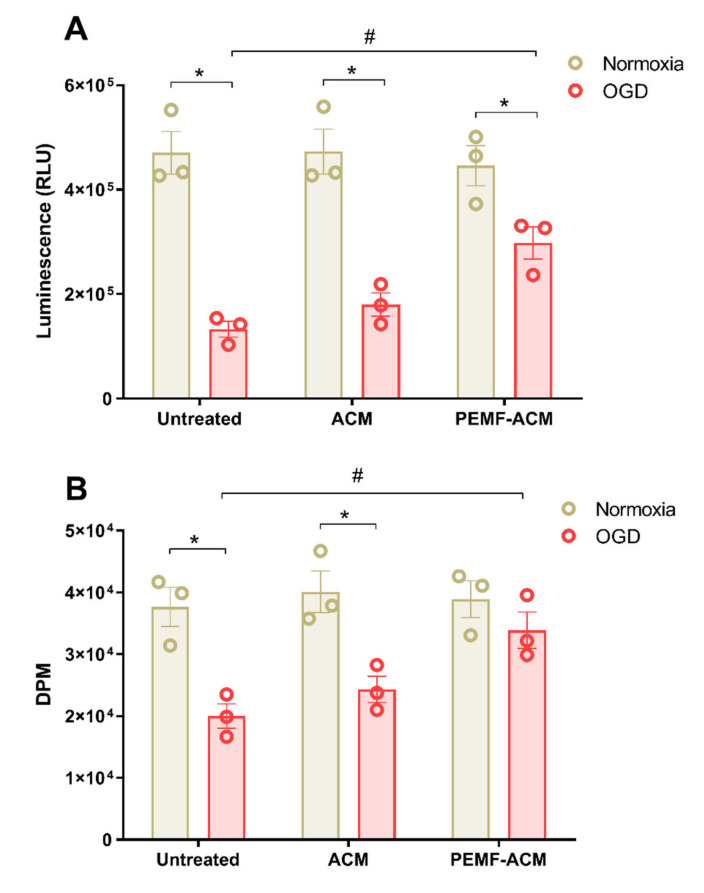
ACM derived from 1321N1 cells exposed to PEMFs confer protection to SH-SY5Y cells from OGD. (**A**) Luminescence expressed as relative light unit (RLU) proportional to ATP intracellular content as an index of SH-SY5Y cell viability. SH-SY5Y were cultured in normoxia or subjected to OGD in standard medium or the presence of 25% ACM derived from 1321N1 cells unexposed (ACM) and exposed to PEMFs in normoxia (PEMF-ACM). (**B**) Disintegrations per minute (DPM) proportional to the amount of [^3^H]-Thymidine incorporation as an index of SH-SY5Y cell proliferation. SH-SY5Y were cultured in normoxia or subjected to OGD in standard medium or the presence of 25% ACM derived from 1321N1 cells unexposed (ACM) and exposed to PEMFs in normoxia (PEMF-ACM). Data are expressed as the mean ± SEM of three independent experiments. *, *p* < 0.01 vs. normoxia. #, *p* < 0.01 vs. untreated cells (standard medium) subjected to OGD.

VEGF is known to exert a neuroprotective action since it prevents neuron from death under critical conditions such as ischemia, through binding to specific receptors expressed on the surface of neuronal cells. To investigate the potential protective role of PEMF-induced VEGF release, astrocytes conditioned medium (ACM) derived from PEMF-exposed 1321N1 was applied to SH-SY5Y cells subjected to oxygen-glucose deprivation (OGD). SH-SY5Y cells were used as a model of neuronal cells and OGD injury was estimated by means of cell viability and proliferation assays. Culturing SH-SY5Y cells in OGD conditions for 48 h induced a significant reduction (72%) of cell viability (Figure 5A). Cell incubation in OGD conditions in the presence of 25% ACM obtained from 1321N1 cells did not affect SH-SY5Y cell viability with respect to standard medium. Interestingly, treating the cells with 25% ACM derived from PEMF-exposed 1321N1 cells significantly increased SH-SY5Y cell viability in comparison to the standard medium (Figure 5A). 

The same experimental approach was then used for the evaluation of SH-SY5Y cell proliferation. As expected, 48 h of OGD determined a significant reduction of cell proliferation in SH-SY5Y cells. Applying 25% ACM to cells cultured in OGD conditions was not sufficient to restore SH-SY5Y cell proliferation (Figure 5B). However, cells treated with 25% ACM derived from PEMF-exposed 1321N1 cells significantly and completely prevented cell proliferation reduction, suggesting its protective effect (Figure 5B).

## 3. Discussion

Emerging evidence indicates PEMFs as a potential strategy for ischemic stroke for their neuroprotective properties [7,8,27]. An open-label, one arm, dose-escalation, exploratory study has established the safety and tolerability of PEMFs in patients with acute ischemic stroke [28]. Recently, the mechanisms underlying PEMF-associated neuroprotective effects were deeply investigated in neuron-like cells [9,10] and microglial cells [11]. However, the effect of PEMF on astrocytes has not been yet investigated. In ischemic stroke, the predominant function of astrocytes is to protect the brain by driving the system back to homeostasis after injury mainly by exerting anti-excitotoxic effects and releasing neurotrophic and angiogenic factors [17,29]. 

The present study revealed a new molecular mechanism by which PEMFs could exert their neuroprotective effects. In 1321N1 cells, used as a model of astrocytes, exposure to PEMFs promoted the release of VEGF, an angiogenic factor with neuroprotective and neurotrophic effects. Indeed, various works in the literature have demonstrated the neuroprotective action of VEGF during cerebral ischemia injury [22,30,31]. VEGF administration later than 24 h after stroke onset seems to always lead to neuroprotection, increased vascular volume, decreased lesion volume, enhanced neural cell proliferation and even improved behavioral recovery from stroke [32]. Importantly, the route of VEGF administration has been indicated as an important factor to consider, since intravenous injection could increase blood-brain barrier (BBB) leakage, while intracerebroventricular application protects the brain against ischemia without adversely affecting BBB permeability [33]. The use of a non-invasive biophysical stimulus represented by PEMFs could imply the advantage of increasing VEGF release directly from astrocytes, possibly avoiding the adverse effects observed with VEGF systemic administration. PEMF-induced VEGF release from astrocyte was similar to that obtained incubating the 1321N1 cells in hypoxic conditions. It is widely accepted that VEGF and other angiogenic and neurotrophic factors such as EPO and TGF-β1 are released in the brain in response to hypoxia and contribute to the adaptive strategy to cope with hypoxic stress [34]. In 1321N1 cells, PEMFs selectively increased VEGF production without affecting EPO or TGF-β1 release. In hypoxic conditions, only EPO levels were increased while TGF-β1 levels were similar to normoxic conditions. 

To better understand the mechanism by which PEMFs promoted VEGF release, the involvement of HIF-1α was investigated. HIF-1α is the main transcriptional activator that function as master regulators of oxygen homeostasis and its stabilization in hypoxic conditions leads to HIF-1-dependent expression of downstream target genes, encoding multiple angiogenic growth factors such as VEGF [35]. Nevertheless, some studies showed that HIF-1α may contribute to cellular and tissue damage stimulating pro-apoptotic molecules that lead to mitochondrial dysfunction and neuronal cell death [36,37]. The treatment of 1321N1 cells with the HIF-1α inhibitor chetomin resulted in a significant reduction of hypoxia-mediated VEGF release, confirming the activation of the HIF-1α pathway. Interestingly, PEMF-mediated VEGF production from 1321N1 cells was not decreased in the presence of chetomin, suggesting a HIF-1α-independent VEGF release. To corroborate this HIF-1α-independent mechanism exerted by PEMF, flow cytometry experiments revealed that PEMFs exposure did not induce HIF-1α expression in 1321N1 cells.

Astrocytes are the most abundant cell type in the central nervous system and play important roles in supporting neuronal cell functions by releasing neurotrophic factors and maintaining homeostasis [38]. ACM represents a widely used in vitro model to test the protective effect of astrocyte-released factors on cultured neurons. In particular, it has been shown that ACM can promote the survival and functional recovery of neurons following different types of injury [39,40,41]. Since PEMFs exposure promoted the production of the neuroprotective factor VEGF, we tested the hypothesis that ACM obtained from PEMF-treated 1321N1 cells could protect SH-SY5Y, an extensively used model of neuron cells, from OGD-induced injury. Our results showed that contrary to control ACM, ACM obtained in the presence of PEMFs significantly prevented cell viability and proliferation decrease induced by OGD in SH-SY5Y cells.

In conclusion, the data obtained in this study provide evidence that PEMFs can influence astrocytes physiology by increasing VEGF release with a HIF-1α-independent mechanism. As indicated by the protective effect on SH-SY5Y subjected to OGD, this effect of PEMFs could be potentially exploited as a non-invasive alternative strategy during cerebral ischemia. The results of the present study add up to those previously published on neuron-like and microglial cells and contribute to defining the mechanism underlying the neuroprotective effect of PEMFs.

## 4. Materials and Methods

### 4.1. Cell Culture and Treatment

The human astrocytoma 1321N1 cell line (Sigma-Aldrich, St. Louis, MO, USA) was maintained in DMEM (Invitrogen, Grand Island, NY, USA) supplemented with 10% FBS (Thermo Scientific, Waltham, MA, USA), L-glutamine (2 mM), penicillin (100 U/mL), and streptomycin (100 µg/mL) in a humidified atmosphere (5% CO_2_) at 37 °C. SH-SY5Y were purchased from American Type Culture Collection (Manassas, VA, USA) and maintained in a 1:1 mixture of Eagle’s Minimum Essential Medium and F12 Medium supplemented with 10% FBS, penicillin (100 U/mL), and streptomycin (100 µg/mL) at 37 °C in a humidified atmosphere with 5% CO_2_ [9].

Hypoxic conditions were obtained culturing the cells in a MiniGalaxy incubator (RSBiotech, Irvine, Scotland) in a humidified atmosphere with 1% O_2_, 5% CO_2_, and balance N_2_ at 37 °C for the indicated length of time.

For the OGD treatment, SH-SY5Y cells were incubated with glucose-free medium in a MiniGalaxy incubator (RS Biotech, Irvine, UK) in a humidified atmosphere with 0.1% O_2_, 5% CO_2_, and balance N_2_ at 37 °C.

### 4.2. Pulsed Electromagnetic Field Exposure System

1321N1 cells were exposed to PEMFs generated by a pair of rectangular horizontal coils (14 cm × 23 cm), each made of 1400 turns of copper wire placed opposite to each other. The culture was placed between this pair of coils so that the plane of the coils was perpendicular to the culture flasks. The coils were powered by the PEMF generator system (IGEA, Carpi, Italy), which produced a pulsed signal with the following parameters: pulse duration of 1.3 ms and frequency of 75 Hz, yielding a 0.1 duty cycle [9,10,11,42]. The peak intensity of the magnetic field and peak intensity of the induced electric voltage were detected in the air between two coils from one side to the other, at the level of the culture flasks. The peak values measured between two coils in the air had a maximum variation of 1% in the whole area in which the culture flasks were placed. The peak intensity of the magnetic field was 1.5 ± 0.2 mT and it was detected using the Hall probe (HTD61-0608-05-T, F.W. Bell, Sypris Solutions, Louisville, KY, USA) of a gaussmeter (DG500, Laboratorio Elettrofisico, Milan, Italy) with a reading sensitivity of 0.2%. The corresponding peak amplitude of the induced electric voltage was 2.0 ± 0.5 mV. It was detected using a standard coil probe (50 turns, 0.5 cm internal diameter of the coil probe, 0.2 mm copper diameter) and the temporal pattern of the signal was displayed using a digital oscilloscope (Le Croy, Chestnut Ridge, NY, USA). The shape of the induced electric voltage and its impulse length were kept constant.

### 4.3. Cell Viability Assessment

Cell viability was evaluated using ATPlite 1 step assay (Perkin Elmer), a system based on the production of light caused by the reaction of ATP with luciferase and D-luciferin [10]. Cells were incubated in the different experimental conditions in black 96-well microplates. At the end of incubation periods, 100 µL of the ATPlite one-step reagent were added to the cells and the microplates were shaken for 2 min at 700 rpm using an orbital microplate shaker. Luminescence was measured with the multimodal plate reader EnSight (Perkin Elmer).

### 4.4. Cell Proliferation Assay

Cell proliferation was evaluated employing [^3^H]-Thymidine incorporation. Briefly, SH-SY5Y cells were seeded (10^5^/cells per well) in fresh medium with 1 µCi/mL [3H]-Thymidine for 24 h. After 24 h of labeling, cells were trypsinized, dispensed in four wells of a 96-well plate, and filtered through Whatman GF/C glass fiber filters using a Micro-Mate 196 cell harvester (Perkin Elmer). The filter-bound radioactivity was counted on a Top Count Microplate scintillation counter with Micro Scint 20 [43].

### 4.5. AlphaLISA Assays

VEGF, TGFβ1, and EPO levels, secreted in the medium by treated and untreated 1321N1 cells, were quantified by using specific AlphaLISA Detection Kits (Perkin Elmer, Boston, MA, USA) following the manufacturer’s instructions. At the end of the experiments, plates were read with the Perkin Elmer EnSight Multimode Plate Reader [11].

### 4.6. Flow Cytometry Analysis

For HIF-1α expression determination, 1321N1 cells were exposed to PEMFs or CoCl_2_ for 24 h. After incubation, cells were rapidly harvested and fixed with 500 µL of intracellular fixation buffer (Invitrogen, cat no. 00-8222). Cells were then incubated for 30 min at room temperature in the dark and centrifuged at 500× *g* for 5 min. The resulting pellet was suspended in 1 mL of ice-cold 90% methanol to permeabilize the cells. After incubation of at least 30 min at 4 °C, cells were washed with an excess volume of flow cytometry staining buffer (Invitrogen, cat n. 00-4222). Aliquots containing 1 × 10^6^ cells were stained with the human HIF-1α directly PE-conjugated monoclonal antibody (clone Mgc3, Invitrogen, cat. n. 12-7528-82) for 45 min at room temperature. Data were acquired on an Attune NxT Flow Cytometer equipped with a 488 nm laser for excitation and fluorescence emission was collected using a 574/26 BP filter. 1321N1 cells were gated according to physical parameters and cell aggregates were removed from the analysis.

### 4.7. Statistical Analysis

Data were analyzed and plotted by using Graphpad Prism (Graphpad Software, La Jolla, CA, USA). Statistical significances were assessed by ANOVA followed by Bonferroni multiple comparison test. All data are reported as the mean ± standard error of the mean (SEM) and differences between conditions were considered significant at *p* < 0.01.

## Abbreviations

PEMFs	Pulsed Electromagnetic Fields
VEGF	Vascular endothelial growth factor
EPO	Erythropoietin
TGF-β1	Transforming growth factor-beta 1
ACM	Astrocyte conditioned medium
OGD	Oxygen-glucose deprivation
BBB	Blood-brain barrier
